# Association of *TRDMT1* Gene Polymorphisms With Neuroblastoma Susceptibility: Insights From a Case–Control Study

**DOI:** 10.1111/jcmm.70848

**Published:** 2025-09-22

**Authors:** Mengzhen Zhang, Wenli Zhang, Chunlei Zhou, Jiaming Chang, Jiabin Liu, Lei Lin, Xinxin Zhang, Liping Chen, Jing He, Baowei Han

**Affiliations:** ^1^ Department of Pediatric Surgery, Guangzhou Institute of Pediatrics, Guangdong Provincial Key Laboratory of Research in Structural Birth Defect Disease, Guangzhou Women and Children's Medical Center Guangzhou Medical University Guangzhou Guangdong China; ^2^ Department of Pathology Children's Hospital of Nanjing Medical University Nanjing Jiangsu China; ^3^ Department of General Surgery Luoyang Maternal and Child Health Hospital Luoyang Henan China

**Keywords:** m5C modification, neuroblastoma, polymorphism, susceptibility, *TRDMT1*

## Abstract

Neuroblastoma is the most common extracranial solid tumour in children, and genetic susceptibility plays a crucial role in its development. The impact of tRNA Dimethyltransferase 1 (TRDMT1), a primary methyltransferase catalysing 5‐methylcytosine (m5C) RNA modification, on neuroblastoma susceptibility remains unexplored. We conducted a case–control study involving 402 neuroblastoma patients and 473 controls from Jiangsu, China. *TRDMT1* polymorphisms (rs7074891 T>C, rs10904887 T>C and rs2273734 C>T) were genotyped via the TaqMan assay. Logistic regression was used to assess odds ratios (ORs) and 95% confidence intervals (CIs), while stratification analysis and expression quantitative trait locus (eQTL) analysis were used to examine subgroup‐specific effects and regulatory impacts. Additionally, clinical correlation analysis and survival analysis were performed on neuroblastoma datasets used to evaluate. The rs7074891 TC/CC genotype reduced neuroblastoma risk (adjusted OR = 0.75, 95% CI = 0.57–0.98, *p* = 0.036), especially in children aged ≤ 18 months and those with mediastinal‐origin tumours. Conversely, the rs10904887 CC (adjusted OR = 1.73, 95% CI = 1.27–2.38, *p* = 0.0006) and rs2273734 TT genotypes (adjusted OR = 1.80, 95% CI = 1.09–2.97, *p* = 0.023) were associated with increased risk, with distinct subgroup‐specific effects. Combined 1–3 risk genotypes further confirmed increased susceptibility (adjusted OR = 1.81, 95% CI = 1.38–2.37, *p* < 0.0001), particularly in males and older children. eQTL analysis revealed that the rs7074891 C and rs10904887 C alleles increased *TRDMT1* expression, whereas the rs2273734 T allele decreased it. Elevated *TRDMT1* expression was correlated with poor prognosis and high‐risk clinical features. *TRDMT1* polymorphisms are significantly associated with neuroblastoma susceptibility, providing insights into their genetic and epigenetic mechanisms and potential as biomarkers and therapeutic targets.

**Abbreviations**
AOR: adjusted odds ratio
CI: confidence interval
COG: Children's Oncology Group
EFS: event‐free survival
eQTL: expression quantitative trait loci
GTEx: genotype‐tissue expression
GWAS: genome‐wide association study
HWE: Hardy–Weinberg equilibrium
INSS: International Neuroblastoma Staging System
m5C: 5‐methylcytosine
OR: odds ratio
OS: overall survival
SNP: single‐nucleotide polymorphism
TRDMT1: tRNA Dimethyltransferase 1

## 1 Introduction

Neuroblastoma is the most common extracranial solid tumour in children, accounting for 8% of all paediatric malignancies and resulting in a mortality rate of 15% [1]. This type of tumour exhibits high clinical and molecular heterogeneity, can arise in any part of the sympathetic nervous system, and displays distinct gene expression profiles among different patients [2]. Clinically, neuroblastoma patients are classified into low‐, intermediate‐, and high‐risk groups based on factors including age at diagnosis, disease stage, histopathological characteristics, *MYCN* amplification, and other genetic markers [3]. While low‐ and intermediate‐risk patients typically achieve an overall survival rate exceeding 90% through surgery and limited chemotherapy [4], high‐risk neuroblastoma remains challenging, with survival rates below 50% and a high relapse risk, highlighting the need for more intensive and personalised treatment strategies [5]. In recent years, an increasing number of genetic polymorphisms and biomarkers have been identified, contributing to risk stratification and offering valuable insights for personalised treatment strategies. For example, genetic alterations such as *MYCN* amplification, *ALK* mutations, *TP53* mutations and *ATRX* mutations have been shown to be closely associated with high‐risk neuroblastoma [6, 7, 8]. Further exploration of the genetic and molecular mechanisms underlying neuroblastoma is crucial for identifying novel therapeutic targets and improving the prognosis of high‐risk patients.

Genome‐wide association studies (GWASs) have emerged as powerful tools for identifying genetic risk factors by analysing millions of single nucleotide polymorphisms (SNPs) across the genome in large‐scale case–control cohorts [9, 10]. Several key susceptibility loci have been identified, with significant associations reported for variants in genes such as *BARD1*, *TP53*, *CASC15*, *CHEK2*, *AXIN2*, *LIN28B*, and *LMO1*, all of which are implicated in tumorigenesis through pathways regulating DNA damage repair, cell proliferation, and apoptosis [11, 12]. Despite these advancements, the genetic landscape of neuroblastoma remains incompletely understood, with ethnic‐specific variations and polygenic risk factors yet to be fully elucidated. Leveraging GWAS findings in combination with functional genomics and epigenetic profiling could provide deeper insights into the molecular mechanisms driving neuroblastoma and pave the way for personalised risk prediction, targeted therapies, and early intervention strategies.

5‐methylcytosine (m5C) is a critical epigenetic modification of RNA that is, catalysed primarily by methyltransferases such as the *NSUN* family and the tRNA Dimethyltransferase 1 (TRDMT1) enzyme [13]. This modification involves the addition of a methyl group (‐CH3) to the fifth carbon atom (C5) of cytosine residues in RNA molecules [14]. m5C modification enhances RNA stability, regulates protein synthesis by influencing ribosome binding efficiency [15, 16], facilitates RNA transport from the nucleus to the cytoplasm, and participates in cellular response to environmental changes, such as oxidative stress and heat shock [17]. Our previous studies demonstrated a significant association between polymorphisms in m5C‐related genes and the risk of neuroblastoma. Specifically, polymorphisms such as rs3998860 G>A and rs12781492 A>C in the *TET1* gene [18], rs10007915 G>C and rs7670522 A>C in the *TET2* gene [19], rs828867 G>A in the *TET3* gene [20], and rs13181449 C>T in the *NSUN2* gene [21] have been identified as closely linked to neuroblastoma susceptibility. These genetic polymorphisms may influence m5C modification levels, thereby regulating gene expression and contributing to the onset and progression of neuroblastoma.

As a key enzyme in m5C modification, the *TRDMT1* gene (also known as *DNMT2*) plays a crucial role in maintaining the dynamic balance of RNA methylation [22]. TRDMT1 mainly catalyses the m5C modification of tRNAs and plays an important role in the cellular stress response and gene expression regulation by modulating RNA stability, translocation and function [23, 24]. Studies have shown that TRDMT1 dysfunction is closely associated with the development of various diseases, including metabolic disorders, viral infections, and certain types of cancer [25, 26]. Specifically, in hepatocellular carcinoma, DNMT2 promotes tumour progression and enhances bortezomib resistance by repressing the pro‐apoptotic gene *TNFSF10* [27]; in colorectal cancer, a TRDMT1‐regulated tRNA fragment tRF‐3022b modulates apoptosis and M2 macrophage polarisation [28]. Clinically, TRDMT1 has emerged as a promising anticancer target, with recent studies identifying small‐molecule inhibitors that disrupt its m5C methylation activity and suppress tumour growth [29]. Given its pivotal role in m5C modification and implication in tumorigenesis, investigating *TRDMT1* gene polymorphisms in neuroblastoma may help elucidate its contribution to tumour susceptibility. This study reveals significant associations between *TRDMT1* gene polymorphisms and neuroblastoma susceptibility, suggesting their potential value as biomarkers for genetic risk assessment.

## 2 Materials and Methods

### 2.1 Study Subjects

This study was approved by the Institutional Review Board of the Children's Hospital of Nanjing Medical University (Approval No. 202112141‐1). Initially, a total of 402 neuroblastoma patients and 473 controls were recruited from the Children's Hospital of Nanjing Medical University in Jiangsu Province, China [21, 30, 31]. The demographic characteristics, including age, sex, and tumour site, are provided in Table S1. There were no significant differences in age or sex distribution between the cases and controls (*p* > 0.05). All participants signed the informed consent form and agreed to participate in the study.

### 2.2 SNP Selection and Genotyping

In this study, three *TRDMT1* SNPs (rs7074891 T>C, rs10904887 T>C and rs2273734 C>T) were selected for analysis. Genotyping was performed according to the method previously published by our team [32, 33, 34]. These SNPs may affect miRNA binding sites, influence RNA splicing, and regulate gene expression levels, as predicted by SNPinfo (https://snpinfo.niehs.nih.gov/snpinfo/snpfunc.html). Genomic DNA was extracted from peripheral blood samples of the control group and children diagnosed with neuroblastoma using the TIANamp Genomic DNA Kit (TianGen Biotech Co. Ltd., Beijing, China). The genotyping of *TRDMT1* gene SNPs was subsequently performed using TaqMan Genotyping PCR PreMix (TianGen Biotech Co. Ltd., Beijing, China). To ensure the accuracy of genotyping, 10% of the samples were randomly selected for replicate testing, and the results demonstrated 100% consistency.

### 2.3 Statistical Analysis

In this study, the chi‐square test and *t*‐test were used to assess significant differences between neuroblastoma patients and the control group. Hardy–Weinberg equilibrium (HWE) for each SNP in the control group was evaluated using the goodness‐of‐fit chi‐square test. Unconditional logistic regression was performed to analyse the associations between *TRDMT1* gene SNPs and neuroblastoma risk, with odds ratios (ORs) and 95% confidence intervals (CIs) adjusted for age and sex. The correlation between SNPs and gene expression was evaluated using data from the Genotype‐Tissue Expression (GTEx) project (https://www.gtexportal.org). Furthermore, the neuroblastoma datasets GSE49710 and GSE45547 were obtained from the R2 Platform (https://hgserver1.amc.nl/). Survival analysis was conducted via the R2 platform to evaluate the impact of *TRDMT1* expression on neuroblastoma prognosis [19]. Kaplan–Meier survival curves were generated to compare survival outcomes among groups with different *TRDMT1* expression levels, and statistical significance was assessed using the log‐rank test. A clinical correlation analysis was conducted to examine the associations between *TRDMT1* expression levels and clinical characteristics, including age, sex, *MYCN* amplification status, Children's Oncology Group (COG) risk classification, and International Neuroblastoma Staging System (INSS) stage. The chi‐square test was used to compare differences between the high‐ and low‐expression groups, with statistical significance set at *p* < 0.05. Data visualisation was performed using R (version 4.4.2) with the ggplot2 package.

## 3 Results

### 3.1 *TRDMT1* Polymorphisms Are Associated With Neuroblastoma Risk

Genotyping of the *TRDMT1* gene was successfully conducted in 394 neuroblastoma patients and 473 control samples among the 402 neuroblastoma patients and 473 controls. The genotype distributions of the three *TRDMT1* SNPs conformed to Hardy–Weinberg equilibrium (HWE, *p* = 0.927 for rs7074891 T>C, *p* = 0.818 for rs10904887 T>C, *p* = 0.594 for rs2273734 C>T; Table 1). The rs7074891 T>C variant was significantly associated with a reduced risk of neuroblastoma in the TC genotype (TC vs. TT: adjusted OR = 0.69, 95% CI = 0.52–0.92, *p* = 0.012). In the dominant model (TC/CC vs. TT), the combined analysis of the TT and TC genotypes also revealed a significant association with reduced disease risk (adjusted OR = 0.75, 95% CI = 0.57–0.98, *p* = 0.036). For the rs10904887 T>C variant, the CC genotype was significantly associated with an increased risk of neuroblastoma (CC vs. TT: adjusted OR = 1.53, 95% CI = 1.06–2.20, *p* = 0.023). According to the recessive model (CC vs. TC/TT), CC genotype carriers were significantly increasing the risk of neuroblastoma (adjusted OR = 1.73, 95% CI = 1.27–2.38, *p* = 0.0006). Furthermore, the rs2273734 C>T variant showed that the TT genotype was significantly associated with an increased risk of neuroblastoma (TT vs. CC: adjusted OR = 1.72, 95% CI = 1.03–2.87; *p* = 0.040). In the recessive model (TT vs. CC/CT), the TT genotype was also linked to a significantly greater disease risk (adjusted OR = 1.80, 95% CI = 1.09–2.97, *p* = 0.023). The combined risk genotype analysis revealed that individuals carrying 1–3 risk genotypes had a significantly increased risk of disease compared with those with 0 risk genotypes (adjusted OR = 1.81, 95% CI = 1.38–2.37, *p* < 0.0001), suggesting a cumulative effect of these SNPs on disease susceptibility.

**TABLE 1:** Association of *TRDMT1* gene polymorphisms with neuroblastoma risk in children from Jiangsu province.

Genotype	Cases (*N* = 394)	Controls (*N* = 473)	*p* ^a^	Crude OR (95% CI)	*p*	Adjusted OR (95% CI)^b^	*p* ^b^
rs7074891 T>C (HWE = 0.927)							
TT	203 (51.52)	210 (44.40)		1.00		1.00	
TC	141 (35.79)	211 (44.61)		**0.69 (0.52–0.92)**	**0.012**	**0.69 (0.52–0.92)**	**0.012**
CC	50 (12.69)	52 (10.99)		1.00 (0.65–1.53)	0.981	0.99 (0.64–1.53)	0.978
Additive			0.244	0.89 (0.73–1.08)	0.244	0.89 (0.73–1.08)	0.242
Dominant	191 (48.48)	263 (55.60)	0.037	**0.75 (0.58–0.98)**	**0.037**	**0.75 (0.57–0.98)**	**0.036**
TT/TC	344 (87.31)	421 (89.01)		1.00		1.00	
CC	50 (12.69)	52 (10.99)	0.440	1.18 (0.78–1.78)	0.440	1.18 (0.78–1.78)	0.441
rs10904887 T>C (HWE = 0.818)							
TT	120 (30.46)	145 (30.66)		1.00		1.00	
TC	158 (40.10)	236 (49.89)		0.81 (0.59–1.11)	0.187	0.81 (0.59–1.11)	0.187
CC	116 (29.44)	92 (19.45)		**1.52 (1.06–2.20)**	**0.024**	**1.53 (1.06–2.20)**	**0.023**
Additive			0.042	**1.21 (1.01–1.45)**	**0.043**	**1.21 (1.01–1.45)**	**0.042**
Dominant	274 (69.54)	328 (69.34)	0.950	1.01 (0.76–1.35)	0.950	1.01 (0.76–1.35)	0.946
TT/TC	278 (70.56)	381 (80.55)		1.00		1.00	
CC	116 (29.44)	92 (19.45)	0.0006	**1.73 (1.26–2.37)**	**0.0006**	**1.73 (1.27–2.38)**	**0.0006**
rs2273734 C>T (HWE = 0.594)							
CC	218 (55.33)	262 (55.39)		1.00		1.00	
CT	136 (34.52)	183 (38.69)		0.89 (0.67–1.19)	0.438	0.89 (0.67–1.19)	0.441
TT	40 (10.15)	28 (5.92)		**1.72 (1.03–2.87)**	**0.040**	**1.72 (1.03–2.87)**	**0.040**
Additive			0.324	1.11 (0.90–1.37)	0.324	1.11 (0.90–1.37)	0.322
Dominant	176 (44.67)	211 (44.61)	0.986	1.00 (0.77–1.31)	0.986	1.00 (0.77–1.31)	0.982
CC/CT	354 (89.85)	445 (94.08)		1.00		1.00	
TT	40 (10.15)	28 (5.92)	0.021	**1.80 (1.09–2.97)**	**0.022**	**1.80 (1.09–2.97)**	**0.023**
Combine risk genotypes^c^							
0	194 (49.24)	301 (63.64)		1.00		1.00	
1–3	200 (50.76)	172 (36.36)	< 0.0001	**1.80 (1.37–2.37)**	**< 0.0001**	**1.81 (1.38–2.37)**	**< 0.0001**

*Note:* Values were in bold if the *p* < 0.05 or 95% CI excluding 1.00.

Abbreviations: CI, confidence interval; HWE, Hardy–Weinberg equilibrium; OR, odds ratio.

^a^ *χ* ^2^ test for genotype distributions between neuroblastoma patients and cancer‐free controls.

^b^ Adjusted for age and sex.

^c^ Risk genotypes were carriers with rs7074891 CC, rs10904887 CC and rs2273734 TT genotypes.

### 3.2 Stratification Analysis of *TRDMT1* Genotypes and Neuroblastoma Susceptibility

We further analysed the associations between the rs7074891, rs10904887, and rs2273734 genotypes and neuroblastoma risk in subgroups stratified by age, sex, site of origin, and clinical stage (Table 2). Stratification analysis revealed that the rs7074891 TC/CC genotype was significantly associated with a lower risk of neuroblastoma in children aged ≤ 18 months (adjusted OR = 0.61, 95% CI = 0.38–0.98, *p* = 0.039) and in those with tumours originating from the mediastinum (adjusted OR = 0.59, 95% CI = 0.39–0.89, *p* = 0.013). For individuals with the rs10904887 CC genotype, several subgroups were significantly associated with an increased risk of neuroblastoma: children aged > 18 months (adjusted OR = 1.72, 95% CI = 1.17–2.54, *p* = 0.006), male sex (adjusted OR = 2.30, 95% CI = 1.50–3.55, *p* = 0.0002), tumours originating from the adrenal gland (adjusted OR = 1.97, 95% CI = 1.21–3.22, *p* = 0.007) and tumours in the retroperitoneal region (adjusted OR = 1.77, 95% CI = 1.18–2.65, *p* = 0.006). Furthermore, individuals carrying the CC genotype of rs10904887 presented a greater neuroblastoma risk regardless of clinical stage, both in early‐stage (I + II + 4s: adjusted OR = 1.98, 95% CI = 1.33–2.94, *p* = 0.0008) and late‐stage patients (III + IV: adjusted OR = 1.62, 95% CI = 1.08–2.45, *p* = 0.021). For the rs2273734 TT genotype, increased neuroblastoma risk was significantly associated with the following subgroups: female sex (adjusted OR = 2.21, 95% CI = 1.06–4.63, *p* = 0.035), tumours originating from the mediastinum (adjusted OR = 2.95, 95% CI = 1.57–5.55, *p* = 0.0008), and patients in early clinical stages (I + II + 4s: adjusted OR = 1.98, 95% CI = 1.07–3.65, *p* = 0.030).

**TABLE 2:** Stratification analysis for the association between *TRDMT1* risk genotypes and neuroblastoma susceptibility in Jiangsu children.

Variables	rs7074891 (cases/controls)		AOR (95% CI)^a^	*p* ^a^	rs10904887 (cases/controls)		AOR (95% CI)^a^	*p* ^a^	rs2273734 (cases/controls)		AOR (95% CI)^a^	*p* ^a^
	TT	TC/CC			TT/TC	CC			CC/CT	TT		
Age, month												
≤ 18	79/64	56/75	**0.61 (0.38–0.98)**	**0.039**	92/109	43/30	1.71 (0.99–2.94)	0.054	120/130	15/9	1.80 (0.76–4.28)	0.180
> 18	124/146	135/188	0.85 (0.61–1.17)	0.313	186/272	73/62	**1.72 (1.17–2.54)**	**0.006**	234/315	25/19	1.77 (0.95–3.29)	0.071
Sex												
Females	96/102	94/123	0.81 (0.55–1.20)	0.294	144/179	46/46	1.24 (0.78–1.98)	0.359	169/213	21/12	**2.21 (1.06–4.63)**	**0.035**
Males	107/108	97/140	0.70 (0.48–1.01)	0.058	134/202	70/46	**2.30 (1.50–3.55)**	**0.0002**	185/232	19/16	1.49 (0.75–2.98)	0.259
Sites of origin												
Adrenal gland	48/210	45/263	0.75 (0.48–1.17)	0.202	63/381	30/92	**1.97 (1.21–3.22)**	**0.007**	83/445	10/28	1.90 (0.89–4.07)	0.097
Retroperitoneal	76/210	88/263	0.93 (0.65–1.32)	0.669	115/381	49/92	**1.77 (1.18–2.65)**	**0.006**	154/445	10/28	1.03 (0.49–2.18)	0.930
Mediastinum	66/210	49/263	**0.59 (0.39–0.89)**	**0.013**	86/381	29/92	1.40 (0.87–2.26)	0.170	97/445	18/28	**2.95 (1.57–5.55)**	**0.0008**
Others	12/210	6/263	0.40 (0.15–1.08)	0.071	11/381	7/92	2.63 (0.99–6.98)	0.052	16/445	2/28	2.08 (0.45–9.59)	0.346
Clinical stages												
I + II + 4s	87/210	83/263	0.76 (0.53–1.08)	0.124	116/381	54/92	**1.98 (1.33–2.94)**	**0.0008**	151/445	19/28	**1.98 (1.07–3.65)**	**0.030**
III + IV	77/210	86/263	0.90 (0.63–1.28)	0.545	117/381	46/92	**1.62 (1.08–2.45)**	**0.021**	154/445	9/28	0.90 (0.41–1.96)	0.790

*Note:* Values were in bold if the *p* < 0.05 or 95% CI excluding 1.00.

Abbreviations: AOR, adjusted odds ratio; CI, confidence interval.

^a^ Adjusted for age and sex, omitting the correspondence factor.

### 3.3 Combined Risk Genotypes and Neuroblastoma Susceptibility Across Subgroups

Stratification analysis indicated that carrying 1–3 risk genotypes was significantly associated with an increased risk of neuroblastoma across various subgroups (Table 3). For children aged ≤ 18 months, the adjusted odds ratio (AOR) was 1.75 (95% CI = 1.08–2.84, *p* = 0.023), whereas for children aged > 18 months, the risk was slightly greater (AOR = 1.83, 95% CI = 1.31–2.54, *p* = 0.0004). Males presented a greater neuroblastoma risk (AOR = 2.02, 95% CI = 1.38–2.94, *p* = 0.0003) than females did (AOR = 1.60, 95% CI = 1.08–2.38, *p* = 0.019). In terms of tumour origin, individuals with tumours located in the mediastinum presented the highest risk (AOR = 2.05, 95% CI = 1.36–3.09, *p* = 0.0006), followed by those with tumours in the adrenal gland (AOR = 1.79, 95% CI = 1.14–2.79, *p* = 0.011) and those with retroperitoneal tumours (AOR = 1.71, 95% CI = 1.19–2.45, *p* = 0.003). Furthermore, patients in clinical stages I + II + 4s carrying 1–3 risk genotypes had a significantly increased risk (AOR = 1.90, 95% CI = 1.33–2.71, *p* = 0.0004), as did those in stages III + IV (AOR = 1.56, 95% CI = 1.08–2.23, *p* = 0.017). Overall, our findings suggest that carrying 1–3 risk genotypes is consistently associated with increased neuroblastoma risk across different subgroups, with significant variations in sex, tumour origin, and clinical stage.

**TABLE 3:** Stratification analysis for the association of combined risk genotypes with neuroblastoma susceptibility in Jiangsu children.

Variables	Risk genotypes (cases/controls)		OR (95% CI)	*p* ^a^	AOR (95% CI)^a^	*p* ^a^
	0	1–3				
Age, month						
≤ 18	66/87	69/52	**1.75 (1.08–2.83)**	**0.023**	**1.75 (1.08–2.84)**	**0.023**
> 18	128/214	131/120	**1.83 (1.31–2.54)**	**0.0004**	**1.83 (1.31–2.54)**	**0.0004**
Sex						
Females	99/143	91/82	**1.60 (1.08–2.38)**	**0.019**	**1.60 (1.08–2.38)**	**0.019**
Males	95/158	109/90	**2.01 (1.38–2.94)**	**0.0003**	**2.02 (1.38–2.94)**	**0.0003**
Sites of origin						
Adrenal gland	46/301	47/172	**1.79 (1.14–2.80)**	**0.011**	**1.79 (1.14–2.79)**	**0.011**
Retroperitoneal	83/301	81/172	**1.71 (1.19–2.45)**	**0.004**	**1.71 (1.19–2.45)**	**0.003**
Mediastinum	53/301	62/172	**2.05 (1.36–3.09)**	**0.0006**	**2.05 (1.36–3.09)**	**0.0006**
Others	9/301	9/172	1.75 (0.68–4.49)	0.245	1.77 (0.69–4.56)	0.234
Clinical stages						
I + II + 4s	82/301	88/172	**1.88 (1.32–2.68)**	**0.0005**	**1.90 (1.33–2.71)**	**0.0004**
III + IV	86/301	77/172	**1.57 (1.09–2.25)**	**0.015**	**1.56 (1.08–2.23)**	**0.017**

*Note:* Values were in bold if the *p* < 0.05 or 95% CI excluding 1.00.

Abbreviations: AOR, adjusted odds ratio; CI, confidence interval; OR, odds ratio.

^a^ Adjusted for age and sex, omitting the correspondence factor.

### 3.4 Correlations Between *TRDMT1* Polymorphisms and Gene Expression

**FIGURE 1:**
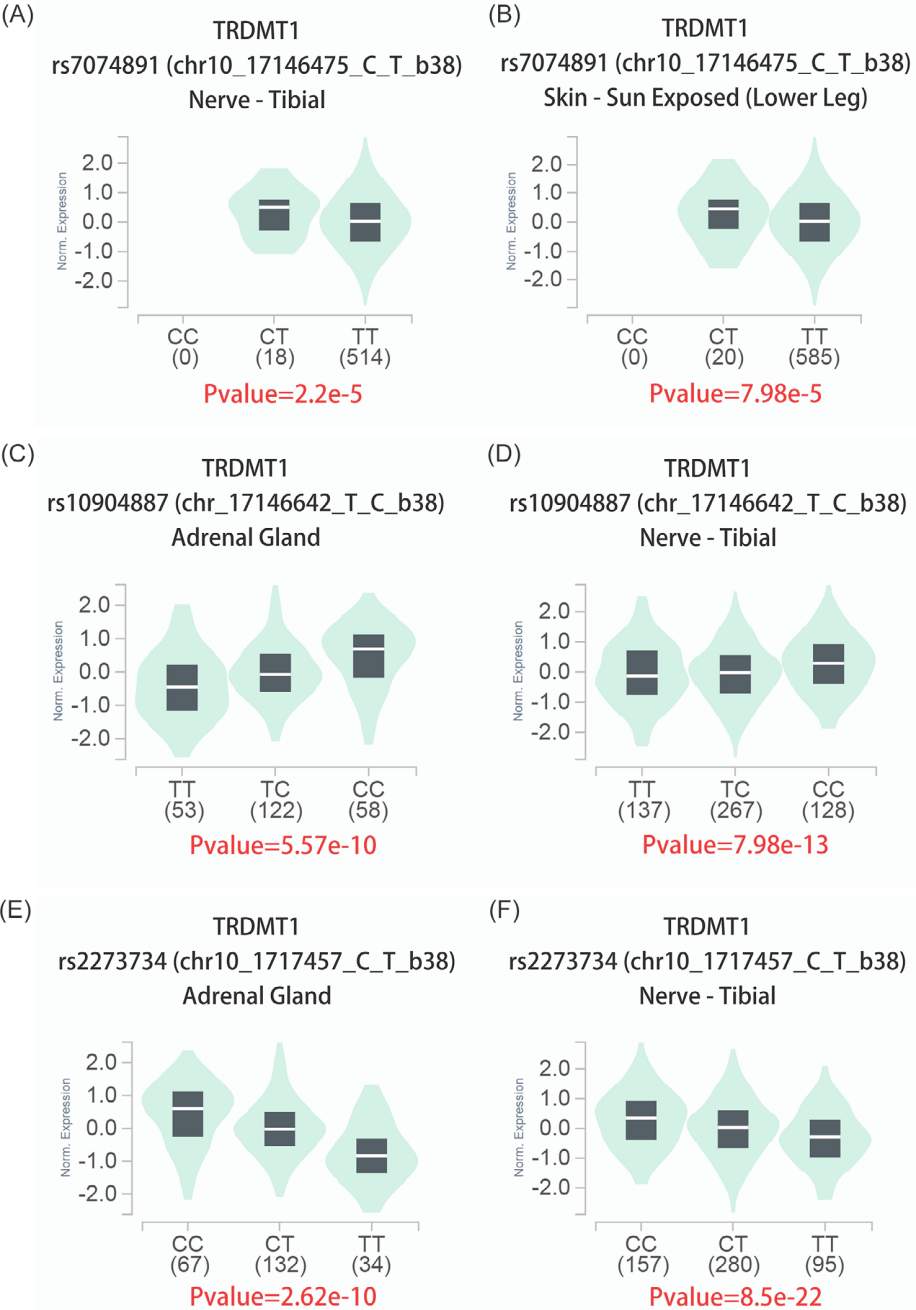
Expression quantitative trait locus (eQTL) analyses of the *TRDMT1* gene polymorphisms rs7074891, rs10904887, and rs2273734 using the genotype‐tissue expression (GTEx) portal database. (A and B) The rs7074891 C allele was significantly associated with increased *TRDMT1* expression in the tibial nerve (*p* = 2.2 × 10^−5^) and sun‐exposed skin (*p* = 7.98 × 10^−5^). (C and D) The rs10904887 C allele is significantly associated with increased *TRDMT1* expression in the adrenal gland (*p* = 5.57 × 10^−10^) and tibial nerve (*p* = 7.98 × 10^−13^). (E and F) The rs2273734 T allele is significantly associated with *TRDMT1* expression in the adrenal gland (*p* = 2.62 × 10^−10^) and tibial nerve (*p* = 8.5 × 10^−22^).

To explore the impact of *TRDMT1* polymorphisms on gene expression, we performed expression quantitative trait locus (eQTL) analysis using the GTEx database. The results demonstrated that the rs7074891 C allele was significantly related to increased *TRDMT1* expression in the tibial nerve (*p* = 2.2 × 10^−5^, Figure 1A) and sun‐exposed skin (lower leg) (*p* = 7.98 × 10^−5^, Figure 1B). Similarly, the rs10904887 C allele was significantly associated with increased *TRDMT1* expression in the adrenal gland (*p* = 5.57 × 10^−10^, Figure 1C) and tibial nerve (*p* = 7.98 × 10^−13^, Figure 1D). In contrast, the rs2273734 T allele was significantly associated with decreased *TRDMT1* expression in both the adrenal gland (*p* = 2.62 × 10^−10^, Figure 1E) and tibial nerve (*p* = 8.5 × 10^−22^, Figure 1F). These findings suggest that *TRDMT1* polymorphisms exert tissue‐specific regulatory effects on gene expression, which may contribute to neuroblastoma susceptibility and progression.

### 3.5 Association of *TRDMT1* With Clinical Characteristics and Prognosis

**FIGURE 2:**
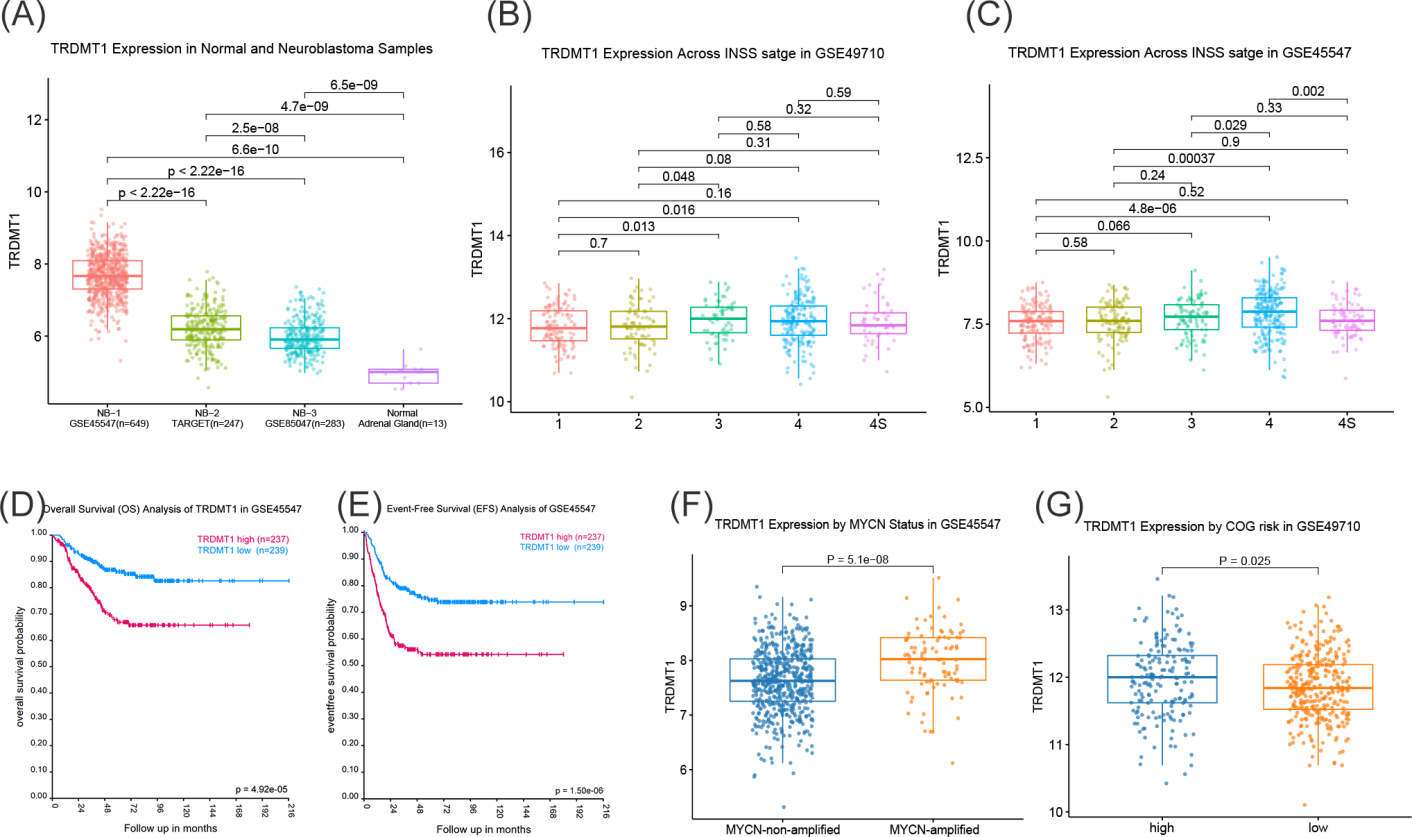
*TRDMT1* expression and its associations with clinical characteristics and prognosis. (A) *TRDMT1* expression levels are significantly lower in normal adrenal gland tissues than in neuroblastoma tissues. (B and C) *TRDMT1* expression varies significantly across International Neuroblastoma Staging System (INSS) stages, with the highest expression in INSS stages III and IV. (D and E) High *TRDMT1* expression is associated with poor overall survival (OS) and event‐free survival (EFS). (F) *TRDMT1* expression levels were significantly greater in the *MYCN*‐amplified group than in the nonamplified group (*p* = 5.1 × 10^−8^). (G) *TRDMT1* expression levels were significantly higher in the high‐risk Children's Oncology Group (COG) group than in the low‐risk COG group (*p* = 0.025).

The expression level of *TRDMT1* in normal adrenal gland tissue was significantly lower than that in neuroblastoma tissue (Figure 2A). Clinical correlation analysis revealed significant differences in *TRDMT1* expression levels across different INSS stages in the GSE49710 and GSE45547 datasets, with higher expression observed in INSS stages III + IV (Figure 2B,C). Survival analysis demonstrated that high *TRDMT1* expression was significantly associated with poorer overall survival (OS) and event‐free survival (EFS) (Figure 2D,E), suggesting that *TRDMT1* may serve as a potential prognostic biomarker for adverse outcomes. Further analysis revealed that *TRDMT1* expression was significantly correlated with *MYCN* amplification status, with higher expression observed in the *MYCN*‐amplified group than in the non‐amplified group (Figure 2F). Moreover, *TRDMT1* expression was significantly higher in the high‐risk COG group than in the low‐risk group (Figure 2G). In summary, these findings suggest that aberrantly high *TRDMT1* expression may play a critical role in the development and progression of neuroblastoma. Its strong associations with tumour stage, prognosis, and risk stratification support its potential as a diagnostic or prognostic biomarker in neuroblastoma.

## 4 Discussion

Neuroblastoma, a highly heterogeneous paediatric malignancy, is influenced by both genetic predisposition and epigenetic regulation [35, 36]. Among RNA modifications, m5C has emerged as a crucial player in tumour development. *TRDMT1*, a key m5C methyltransferase, has been implicated in cancer progression, but its role in neuroblastoma remains undefined.

In this study, we identified associations between *TRDMT1* gene polymorphisms (rs7074891 T>C, rs10904887 T>C and rs2273734 C>T) and neuroblastoma susceptibility in a Chinese paediatric population. Notably, the rs7074891 TC genotype appeared protective, whereas the rs10904887 CC and the rs2273734 TT genotypes conferred elevated risk. These associations were further supported by stratification and combined risk genotype analyses, suggesting that *TRDMT1* genetic variants may contribute to individual differences in neuroblastoma risk.

Aberrant m5C modifications dysregulation of their associated enzymes have been increasingly linked to tumorigenesis, primarily through effects on RNA methylation, transcript stability, and gene expression [23, 37]. Members of the *NSUN* and *TET* families have been extensively studied in this context. For instance, *NSUN2* promotes gastric and bladder cancer progression [38], and its polymorphism rs13181449 C>T is associated with reduced neuroblastoma susceptibility through diminished *NSUN2* expression and lower m5C levels [21]. Likewise, *TET1*–*3* polymorphisms are associated with neuroblastoma risk by maintaining stemness and cellular plasticity through non‐catalytic mechanisms [39, 40].

*TRDMT1* catalyses the m5C modification at cytosine 38 in the tRNA anticodon loop, contributes to RNA stability, translational accuracy, and cellular homeostasis [41, 42]. Its overexpression in hepatocellular and colorectal cancers has been linked to tumour progression and poor prognosis [27, 28], and the rs34434809 G>C polymorphism may lead to worse outcomes in hepatocellular carcinoma [43]. However, the precise role of *TRDMT1* and its polymorphisms in neuroblastoma remains unexplored.

For the first time, we demonstrated that *TRDMT1* SNPs are significantly associated with neuroblastoma susceptibility. The rs7074891 TC genotype provided protective effects against neuroblastoma, particularly in children under 18 months of age and those with tumours originating from the mediastinum. Conversely, the rs10904887 CC and rs2273734 TT genotypes were associated with increased risk in older children and patients with tumours from the adrenal gland or retroperitoneal regions. These heterogeneous effects across age, sex, and tumour origin suggest that the penetrance of *TRDMT1* SNPs may depend on host and tumour context, consistent with findings that genetic variant effects can vary across tissues or developmental states [44, 45].

Further analysis of combined risk genotypes revealed that individuals carrying 1–3 risk genotypes had a significantly increased risk of neuroblastoma across most subgroups. Notably, the effect was more pronounced in males, patients with adrenal or mediastinal tumour origin, and those with advanced clinical stages. These findings suggest a cumulative impact of multiple risk genotypes on disease susceptibility, with stronger effects in specific host backgrounds or different tumour characteristics [46].

eQTL analysis revealed that these SNPs influence *TRDMT1* expression in various tissues, including the adrenal gland, tibial nerve, and skin. Notably, the rs7074891 C and rs10904887 C alleles were associated with increased *TRDMT1* expression, whereas the rs2273734 T allele was linked to reduced expression. These findings suggest that different alleles may modulate neuroblastoma risk by increasing or decreasing *TRDMT1* activity. The cumulative effect of combined risk genotypes further supports the critical regulatory role of these alleles in disease susceptibility, consistent with previous reports highlighting the impact of multi‐locus genetic variants on cancer risk [47].

Survival analysis confirmed that elevated *TRDMT1* expression is associated with poorer prognosis in neuroblastoma patients. Furthermore, increased *TRDMT1* expression is correlated with critical clinical features such as *MYCN* amplification and INSS staging, suggesting its role in tumour progression. These findings collectively suggest that *TRDMT1* may play an oncogenic role in neuroblastoma progression and support its utility as a biomarker for aggressive disease [42].

Overall, our findings identified the associations between m5C key gene *TRDMT1* polymorphisms and neuroblastoma susceptibility, prognosis, and clinical characteristics. Through comprehensive analyses, including stratification analysis, combined risk genotype analysis, eQTL analysis, survival analysis and clinical correlation analysis, our findings highlight *TRDMT1* as a potential marker for neuroblastoma risk assessment and a therapeutic target. However, the study is limited by its relatively small sample size and the lack of validation in an independent cohort, which may reduce the generalisability of the observed association. Future research with larger datasets is needed to validate these findings and further investigate the specific functional mechanisms of these polymorphisms, including their effects on m5C levels, *TRDMT1* enzymatic activity, and downstream RNA stability.

## 5 Conclusion

Our study revealed a significant association between *TRDMT1* gene polymorphisms (rs7074891 T>C, rs10904887 T>C, and rs2273734 C>T) and neuroblastoma susceptibility. These results suggest that *TRDMT1* may serve as a molecular marker for neuroblastoma risk assessment and prognosis.

## Author Contributions

**Mengzhen Zhang:** formal analysis (equal), investigation (equal), writing – original draft (equal), writing – review and editing (equal). **Wenli Zhang:** funding acquisition (equal), investigation (equal), writing – original draft (equal), writing – review and editing (equal). **Chunlei Zhou:** investigation (equal), resources (equal), writing – original draft (equal), writing – review and editing (equal). **Jiaming Chang:** funding acquisition (equal), investigation (equal), writing – review and editing (equal). **Jiabin Liu:** investigation (equal), writing – review and editing (equal). **Lei Lin:** investigation (equal), writing – review and editing (equal). **Xinxin Zhang:** funding acquisition (equal), investigation (equal), writing – review and editing (equal). **Liping Chen:** funding acquisition (equal), investigation (equal), writing – review and editing (equal). **Jing He:** conceptualization (equal), formal analysis (equal), funding acquisition (equal), investigation (equal), supervision (equal), writing – original draft (equal), writing – review and editing (equal). **Baowei Han:** conceptualization (equal), investigation (equal), supervision (equal), writing – review and editing (equal).

## Ethics Statement

The research scheme was approved by the Institutional Review Board of Children's Hospital of Nanjing Medical University (Approval No: 202112141‐1).

## Consent

Written informed consent was obtained from all participants in accordance with the Declaration of Helsinki.

## Conflicts of Interest

The authors declare no conflicts of interest.

## Supporting information


**Table S1:** Demographic characteristics of neuroblastoma patients and cancer‐free controls from Jiangsu province.

## Data Availability

Data supporting the findings of this study are available upon reasonable request.
